# Autoimmunity in Cardiomyopathy-Induced Heart Failure and Cardiac Autoantibody Removal by Immunoadsorption

**DOI:** 10.3390/jcm14030947

**Published:** 2025-02-01

**Authors:** Michael Dandel

**Affiliations:** German Centre for Heart and Circulatory Research (DZHK), Potsdamer Str. 58, 10785 Berlin, Germany; mdandel@aol.com; Tel.: +49-308224210

**Keywords:** dilative cardiomyopathy, heart failure, autoimmunity, cardiac autoantibodies, immunoadsorption, myocardial recovery

## Abstract

There is increasing evidence that β1-adrenoreceptor autoantibody (β1AR-AAb) elimination can break the vicious circle induced by certain pathological conditions associated with alteration of the physiological self-tolerance, followed by generation of such AAbs and activation of cell-mediated immune processes directed against the myocardium. Concerning this, the present narrative review article provides an updated overview of the state of knowledge about the role of auto-immunity in the etiopathogenesis of cardiomyopathies, with a particular focus on immunoadsorption (IA) therapy for β1AR-AAb-positive adult patients with a dilated cardiomyopathy (DCM)-associated refractory heart failure (HF). Among many relevant findings, the increasing prevalence (up to 97%) of β1AR-AAb-positive patients related to the aggravation of HF, the high prevalence (between 84% and 91%) of HF patients in which IA can reduce to a minimum any increased β1AR-AAb level, as well as the high prevalence (about 80%) of responders to the IA-induced normalization of β1AR-AAb levels by long-term improvement in LV ejection fraction with increase in LV stroke volume and cardiac output, are of particular relevance. Given that after the elimination of β1AR-AAbs in potential candidates for heart transplantation (HTx), the post-IA 3- and 5-year HTx-/mechanical support-free survival probability reached 80% and 63-69%, respectively, the good tolerability of IA and the possibility to repeat that therapy also in elderly persons strongly suggest that in appropriately selected patients, this therapy deserves much more attention in the future.

## 1. Introduction

A healthy immune system identifies any possibly detrimental foreign substance invading the organism and coordinates the defense against that immune response stimulating “non-self” substance, called antigen (Ag), in order to minimize its pathogenic potency and prevent any deleterious consequences for the organism [1]. Although the organism’s own substances (e.g., cells, tissues, and macromolecules) are overall excluded from immune system attacks, this self-tolerance can be broken under specific pathological conditions, in which the immune system generates autoantibodies (AAbs) and activates cell-mediated immune processes directed against certain own substances, which, under these circumstances will become “self-Ags”. Such autoimmune responses to self-Ags have an important impact on the development and the severity of specific myocardial diseases, especially dilated cardiomyopathy (DCM), which is among the most common causes of heart failure (HF) [1,2]. Given that in DCM, which is the leading indication for heart transplantation (HTx), the prevalence of functional AAbs against β1-adrenoreceptors (β_1_AR-AAbs) in the sera of patients with end-stage HF before HTx or left ventricular assist device (LVAD) implantation, can exceed average values of 80% and 90%, respectively, these still insufficiently considered findings deserve more attention [3,4,5,6]. One particular reason for the increasing interest is the growing body of evidence, which indicates that the timely inactivation and/or removal of certain types of cardiac AAbs (particularly β1AR-AAbs) can stop the progression of HF and also facilitate reverse myocardial remodeling associated with functional improvement, which can improve the patient’s quality of life, and also provide important survival benefits in most cases [1].

This narrative review article aims to give an updated overview of the state of knowledge about the role of autoimmune mechanisms in the etiopathogenesis of cardiomyopathies (CMs), with a particular focus on immunoadsorption (IA) therapy for β_1_AR-AAB-positive adult patients with DCM-associated refractory HF. Some of the most important novelties that were introduced in this article are the following:

- conclusive evidence for the existence of a positive correlation between the aggravation of DCM-associated HF and the prevalence of functional β1AR-AAb-positive HF patients, which can exceed 95% in HTx candidates;
- high efficiency of IA in the removal of β1AR-AAbs;
- up to 80% prevalence of cardiac responders (i.e., improvement in LV function) among the patients with β1AR-AAb reduction below their pathological level;
- high stability of the reduced post-IA levels appeared to be high even beyond the third post-IA year;
- good tolerability and the possibility of repeating the IA also in elderly HF patients.

## 2. Role of Autoimmune Responses in the Pathogenesis of Heart Failure

Whereas HF is a well-described final common pathway for a wide variety of myocardial disorders, and the diagnosis of obstructive coronary artery disease (CAD) is also well-established, the evidence and evaluation of the usually highly complex underlying etio-pathogenic particularities of non-ischemic myocardial diseases pose special challenges with an important impact on both clinical decision-making and therapeutic success [7,8,9]. Regarding this, the detection of chronic inflammation with autoimmune responses and especially the evaluation of their potential contribution to the severity and the progression rate of myocardial dysfunction can create particular problems.

### 2.1. Myocardial Damages Induced by Infection, Inflammation, and Autoimmunity

The existence of a vicious circle initiated by myocardial injury of various etiologies, followed by cell death, inflammation, T-cell activation, and anti-heart autoimmunity, which, in turn, induces new myocardial injury, which further aggravates myocardial dysfunction responsible for development, persistence, and progression of HF (illustrated in the Graphical Abstract), has been repeatedly demonstrated [7,8,10]. Although the contribution of autoimmunity was found to be much more evident in HF associated with CMs, particularly with idiopathic DCM, there are indicators that suggest a possible contribution of such a self-sustaining vicious circle also in certain patients with ischemic HF [10,11,12,13].

Because a coincidental occurrence of obstructive CAD and idiopathic DCM cannot always be ruled out, since the end of the 1980s, this possibility has generally been accepted [14,15]. In this regard, a necropsy study identified 18 patients with chronic congestive HF, which was not attributable to the existent obstructive CAD but appeared rather related to a coexistent idiopathic DCM [14]. The morphologic myocardial alterations suggested that those patients had rather primary DCM, and the CAD was coincidental [14]. In another earlier necropsy study on 45 deceased patients with previous angiographic evidence of obstructive CAD, 9 (20%) of them additionally revealed morphologic myocardial alterations, which indicated the presence of a coincidental non-ischemic idiopathic DCM [15]. In a recent study, which included 3023 patients with obstructive CAD confirmed by coronary angiography, late gadolinium enhancement cardiac magnetic resonance imaging (LGE-MRI) revealed in 65% of those patients the presence of ischemic CM, in 9% of them the presence of a coincidental nonischemic CM, and in other 8% of them the presence of both ischemic and nonischemic CM (i.e., “dual CM”) [12]. Patients with obstructive CAD plus nonischemic or dual CM showed a worse prognosis compared to those with ischemic CM [12]. These findings suggest that patients with CAD and coincidental non-ischemic or dual CM may have suboptimal outcomes after coronary revascularization [12,13]. These and other similar observations could explain the lack of therapeutic and/or prognostic benefits of coronary revascularization observed in large previous studies [16,17,18], which compared patients with moderate or even severe stable obstructive CAD associated with left ventricular (LV) systolic dysfunction who underwent revascularization, to those maintained on a conservative strategy based on optimal medical therapy [12,13].

Taking the above-mentioned new findings into consideration, the recent suggestion for a reconsideration of ischemic and non-ischemic CM terminology is quite understandable [19]. Although, since 2008, CMP has generally been defined as “myocardial disorder in which the heart muscle is structurally and functionally abnormal in the absence of CAD, hypertension, valvular disease, and congenital heart disease sufficient to cause the observed myocardial abnormality” [20], CMs were, thereafter, in the medical literature furthermore (strikingly often), classified into non-ischemic and ischemic CMs. Thus, in a representative overview of CMs, Braunwald E. [21] further used, in 2017, the designations of ischemic and non-ischemic cardiomyopathies to emphasize the importance of the coronary artery circulation for the distinction between the most frequent myocardial diseases. Even in 2024, more than 15 years since the newest definition of the CM, which included the absence of CAD as a mandatory prerequisite for a CM, the PubMed database cited during the first 9 months a total of 207 articles that used the terms “ischemic cardiomyopathy and/or non-ischemic cardiomyopathy”.

Inflammatory CM, defined as myocarditis associated with both ventricular remodeling and cardiac dysfunction, can have very different causes but is predominantly mediated by viral infection and is mainly characterized by myocardial infiltration with inflammatory cells [22,23,24,25,26,27]. In addition, inflammatory CM can be triggered by bacterial, fungal, or protozoan infections as well as by different drugs, toxic substances, or systemic immune-mediated diseases [22,23,24]. Regarding the role of viral infection, it appears necessary to make a distinction between virus-induced and virus-associated inflammatory CM and between viruses that infiltrate the heart and those that do not necessarily need to invade the cardiac cells because they can also indirectly induce myocardial injury and dysfunction by inducing a cytokine storm or cellular immune responses by molecular mimicry [23].

Viral-induced myocarditis is basically an autoimmune process inducible by a large variety of different types of viruses characterized mainly by a disruption of autoimmune control mechanisms, which results in a specific inflammatory response directed against certain body and viral antigens [28,29]. By myocardial tissue invasion and entering into the cardiomyocytes, the viruses activate different inflammatory mediators via natural killer (NK) cells and macrophages, especially Interleukin-1, tumor necrosis factor-alpha (TNFα), and Interferon-gamma [28,30]. This leads to cardiomyocyte damage and apoptosis with fibrosis at the damaged site and, in a chronic course of virus-induced inflammation and infection, this leads to the development of HF [28]. In the case of chronic HF with subsequent DCM, about 67% are triggered by virus infection [28]. There are about 27 viruses that were found to be directly or indirectly involved in the etiology of myocarditis [31]. Among these, the most common are enteroviruses (Coxsackie A and B viruses, Echoviruses), erythroparvovirus (parvovirus B19), herpes virus (HHV6), Epstein–Barr virus (EBV), cytomegalovirus (CMV), lentivirus (HIV), hepacivirus (HCMV), influenza A and B viruses [32]. Viruses of the Coxsackie species subtype B are generally considered among the most common etiological factors of viral myocarditis, complicated finally by the development of DCM [28]. However, more recently, there have been indications that the parvovirus subtype B19 (PVB19), which is the only erythroparvovirus that has the ability to infect human cells, is possibly even more frequently involved in the development of DCM [28]. As a vasotropic virus, PVB19 does not directly enter the cardiomyocytes, and in the heart, the target points are the endothelial cells of myocardial blood vessels [28,33]. After the disappearance of the primary infection symptoms, the virus enters its latent phase, which is associated with a weakening of the immune system [28].

### 2.2. Importance of Immune Cells in the Pathogenesis of Cardiomyopathies

Immune responses require the participation of different cell types belonging to both innate and adaptive immune cells. The innate immune cells, which include phagocytes (neutrophils and monocytes/macrophages), dendritic cells (DCs), NK cells, gamma delta T cells, NK-T cells, B1 cells, and innate lymphoid cells, are the first responders to infections in all species [34]. By contrast, the distinct roles of the adaptive immune B and T cells, which express distinct antigen receptors necessary to prevent future foreign invasions by developing memory responses and which are also primarily involved in the pathogenesis of autoimmune diseases, were detected only in vertebrates [34].

The importance of immune cells in the pathogenesis of viral inflammatory CM has been unequivocally proven [23,24]. The time course of the pathogenic processes comprises three phases: the first, a short (1 to 7 days long) acute phase, characterized by virus invasion of cardiac cells and activation of innate immune responses; the second, a subacute phase of activation of the adaptive immune responses lasting 1 to 4 weeks; and the third, a chronic phase, which can last months or years, in which delayed or ineffective viral clearance associated with chronic cardiac inflammation and myocardial remodeling can lead to DCM and finally, also to decompensated HF [23].

The innate immune responses to infection are initiated by activation of innate immune cells after the recognition of specific molecular patterns of pathogens and molecular patterns released from endogenous damaged cells by their recognition receptors (e.g., Toll-like receptors) [23]. Both the recognition receptor and the downstream signaling can vary according to the pathogen or the damage-associated molecular patterns [23]. The triggered innate immune cells and cardiac cells release cytokines, chemokines, and interferons, inducing further activation and housing of innate immune cells in the heart, including mast cells, neutrophils, DCs, monocytes, and macrophages [23,35,36,37,38]. Even though such cardiac innate immune responses are basically beneficial due to their antiviral impacts, disproportionate and/or sustained activation of the innate immune system can lead to excessive and/or chronic inflammation that induces myocardial damage and remodeling, which finally results in cardiac dysfunction and eventually HF [23,35]. Studies revealed that, whereas viral infection-induced recruitment of natural killer cells and DCs can prevent the development of myocarditis [23,37,38], mast cells, which are among the first cells involved in the initial innated immune responses by producing proinflammatory cytokines like tumor necrosis factor, interleukin-1β, and interleukin-4 can also have major contributions to the development of autoimmune responses [23,36,37]. Mice susceptible to autoimmune cardiac disease after coxsackie virus B3 (CVB3) infection showed a particularly high number of mast cells [36].

After cardiac viral infection, simultaneously with the monocyte infiltration, the DCs also assemble in the infected myocardium, and after ingesting dead and damaged myocytes, these DCs migrate to regional lymph nodes and the spleen, where they present antigens to naive B cells and T cells, which initiate the activation of adaptive immune response [23,39]. Experimental studies have proven the importance of DCs as Ag-presenting cells by demonstrating that cardiac DC depletion can prevent the generation of antigen-specific CD8^+^ T cells, favoring the worsening of subclinical cardiac morphological and functional alterations toward the end-stage HF [23,39]. DCs can also process endogenous antigens and, therefore, might trigger autoimmune myocarditis [23,39].

Despite being crucial to limiting the myocardium’s viral infection, the host immune response can also become responsible for cardiac damage in predisposed persons [40,41]. Exaggerated leucocyte activation can facilitate severe life-threatening acute myocardial dysfunction and/or severe DCM with progressive development of advanced HF [40,41]. Neutrophils, which are the most abundant circulating immune cells, are also part of the first lines of defense against cardiac infection, and their activation takes place before that of other innate immune cells [23,40]. They can maintain inflammation by a specific process based on the formation of neutrophil extracellular traps (NETs) called NETosis [23,40,41,42]. Experimental neutrophil depletion and NET blockade in the early acute phase of cardiac infection with CVB3 appeared to be able to reduce the cardiac immune response and myocardial necrosis by also influencing monocyte and macrophage function at the site of inflammation [40]. Inhibition of NET formation in mice with autoimmune myocarditis induces a reduction in inflammation in the acute phase of the disease [3]. Targeting the cytokine midkine, which mediates NET formation in vitro, not only attenuates NET formation in vivo and the infiltration of polymorphonuclear neutrophils but also reduces the fibrosis and preserves the myocardial systolic function during experimental autoimmune myocarditis [42]. The NETs can enhance inflammation and tissue injury by different mechanisms, including direct damage, platelet activation, triggering AAb production, and reducing the threshold for T-cell activation [40,42,43,44].

Monocytes and macrophages, a heterogeneous and multifunctional cell population originating from a common myeloid progenitor cell in the bone marrow with an essential role in the pathogenesis of myocarditis, represent a large part of cells infiltrating the damaged heart [23,45]. After the cardiac injury, monocytes and tissue-resident macrophages traverse phenotypic and functional changes, which make possible their function as key regulators of tissue repair, regeneration, and fibrosis [23,43]. Macrophages, which are large phagocytic cells capable of activating and multiplying lymphocytes in order to generate innate and adaptive immune responses, are major regulators of immune systems with an important immunoregulatory role in inflammatory CMs [23,45]. Resident macrophage dysfunctions with uncontrolled production of inflammatory cytokines, inefficient anti-inflammatory responses, and altered communication between macrophages and epithelial cells, endothelial cells, and fibroblasts can lead to persistent injury, altered myocardial repair, and, finally, to HF [40,46]. Thus, when the pro-inflammatory and anti-inflammatory phases are not balanced, it can have detrimental effects on myocardial systolic and diastolic function [23,45].

Given that aberrant B-cell and T-cell reactivity to normal components of the host characterizes all of the very diverse autoimmune diseases and, in this context, antigen-specific CD4+ T helper cells can stimulate AAb production by B cells, whereas cytotoxic CD8+ T cells can damage or kill various other cells, particular attention needs to be paid to T and B cells [47]. Basically, B and T cells mediate their effector functions partly independently and partly cooperatively in removing invading pathogens. At the same time, their self-tolerance enables the identification of self-produced antigens in order to not react against them and, thus, avoid deleterious autoimmune responses [34]. Autoimmune diseases are primarily mediated by adaptive immune cells, and thus, as soon as the self-tolerance is broken, autoimmune responses can be mediated by autoreactive T cells, AAbs, or both [34]. Gene variants can create a predisposition to immune dysregulation, whereas environmental factors appeared also necessary for the induction of T- and/or B-cell autoreactivity and for the clinical manifestations of immune dysregulation [34,47].

In experimental studies, T cells and certain B cells specific for viral antigens were identified as important intermediaries of cardiac damage, which can induce HF [23,27]. T-cell activation is considered an essential pathophysiological mechanism involved in the occurrence of both autoimmune myocarditis and autoimmune inflammatory CM [48]. Self-reactive CD4^+^ IL-3^+^ T cells appeared able to amplify autoimmune inflammation in myocarditis by stimulating monocyte chemotaxis, which additionally recruits monocytes able to differentiate into macrophages and DCs [48]. The process later repeats itself when DCs, upon meeting effector T cells in the tissue, stimulate local T-cell proliferation [48].

B cells are a decisive link between the innate and adaptive immune system [23,49]. In addition to the antigen-specific B-cell receptors, B cells also express toll-like receptors (TLRs) on their cell surface, which are activatable by both exogenous (viral or bacterial) or endogenous antigens and, thereby, represent an indispensable part of the innate immune system [43]. TLR stimulation is associated to both B-cell activation and tolerance [49]. TLR-4 expression was found to be upregulated in human HF [49,50]. There is also evidence that in the myocardial tissue, whereas short-term activation of TLRs confers cyto-protection, long-term activation can cause upregulation of proinflammatory cytokines and recruitment of immunoactive cells like DCs, monocytes, and neutrophils into the myocardium [51].

B-cell depletion therapies by using either monoclonal Abs or a chimeric antigen receptor-based therapy, which are currently approved or employed for the potential treatment of various autoimmune diseases, appeared not suitable for CMs associated with the particular class of AAbs directed against G-protein-coupled receptors (GPCR-AAbs), which also incorporates the β1AR-AAbs [1,52,53]. Concerning Rituximab (i.e., the first agent that was used for selective depletion of CD20-positive B cells), the study’s results regarding its potential usefulness in the treatment of diseases associated with GPCR-AAbs were inconsistent [1]. In addition to those uncertainties about the benefits of its presumed inhibiting impact on the GPCR-AAb-induced adverse cardiac effects, an additionally possible direct cardiotoxic effect of Rituximab cannot be excluded, specifically in patients with already relevantly damaged myocardium [1,54]. 

**Figure 1:**
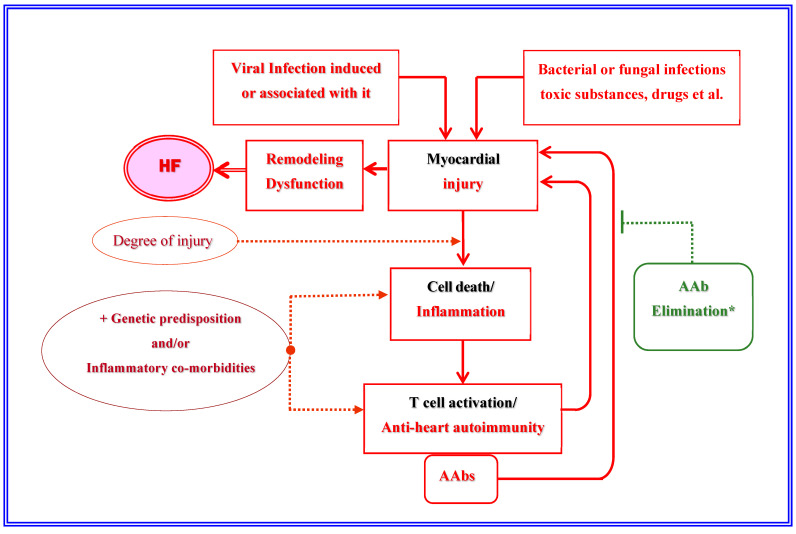
Overview of the major pathophysiologic mechanisms involved in the development of non-ischemic cardiomyopathy-related chronic heart failure. HF: heart failure; AAbs: autoantibodies [1,2,3,4,5,6,7,10,21,22,23,24,27,34,35,36,37,38]; * AAb elimination can break the vicious circle and, thereby, not only stop the progression of HF but can also favor myocardial reverse remodeling and functional improvement.

Figure 1 provides a graphical overview about the main pathophysiologic steps toward the development of inflammatory CM-related chronic HF.

## 3. Cardiotoxic Autoantibodies and Immunoadsorption Therapy

It is well established that normal individuals display circulating immunoglobulins (Igs) able to bind a variety of foreign antigens (mainly bacteria, viruses, and fungi), as well as different self-components (e.g., apoptotic and necrotic cells, cellular components, nucleic acids, phospholipids, and serum proteins) [55]. Because these Abs, which are produced mainly by CD5+ B cells, occur independently of known and/or deliberate immunization, they have been termed natural Abs (NAbs) [56,57]. Given that a significant proportion of the NAb pool interacts with certain self-antigens, they are also called natural autoantibodies (NAAbs) [55]. In contrast to antigen-induced Abs, which are mainly IgG and mono-reactive, a considerable proportion of NAbs are mainly IgM and typically polyreactive (i.e., they bind several unrelated antigens with different affinities) [57]. Because of their broad reactivity for a wide variety of microbial components, which is directly related to the essential biological functions of NAbs (i.e., removal of cellular and molecular waste, targeting of pathogens, as well as immunoregulation, maintenance of the immune system homeostasis and prevention of autoimmunity), NAbs play a major role in the primary line of defense against infections [56]. The immunoregulatory role of NAbs designates them as valuable diagnostic, prognostic, and therapeutic (i.e., use as intravenous Ig preparations) agents [57].

### 3.1. Major Types of Potentially Cardiac Damaging Autoantibodies

Autoimmunity is widely accepted as the origin or amplifier of various diseases subdivided into autoinflammatory diseases and autoimmune diseases, which are characterized by chronic aberrant activation of the immune system, causing tissue inflammation and damage [58]. The pathogenetic mechanisms underlying the tissue damage differ between those two types of diseases. Thus, whereas the innate immune system is directly responsible for tissue inflammation in autoinflammatory diseases, autoimmune diseases occur when a specific adaptive immune response is redirected against self-antigens [58]. Autoimmune responses based on Abs against self-antigens also play a determinant role in the pathogenesis, severity, and prognosis of different cardiovascular diseases, including DCM [57,58]. Depending on the type of the studied AAbs, the method used for their detection, and the severity of HF, cardiac AAbs were detectable in up to 60% of patients with ischemic and up to 100% of those with non-ischemic HF (Table 1) [58,59,60,61,62,63]. Although the emergence of self-reactive endogenous AAbs is still not completely clarified, it is presumed that this can be triggered by tissue injury and/or dysregulated humoral response, mainly in the presence of a genetic predisposition [57,58]. Autoimmune diseases triggered by deleterious autoimmune Ab responses to self-antigens are a large and diverse group of pathological conditions characterized by immune disturbances that cause aberrant B-cell and T-cell reactivity to normal constituents of the host, which also play a determinant pathogenetic role in CMs and other cardiovascular diseases, with detrimental impact on patient outcome [47].

The immune system, which is of vital importance for the recognition and development of appropriate responses aimed to prevent detrimental damages inducible by a wide variety of pathogens that could invade the organism or could be generated inside the body (e.g., cancer cells), is a complex network of highly sensitive biological systems. In contrast to classic AAbs, which induce immune responses resulting in the destruction of the affected tissue, the GPCR-AAbs induce dysfunctions by activation of receptor-mediated signal cascades [1,59,63]. The role of GPCRs is to detect an extracellular agonist, transmit the information across the cell membrane, and activate a cytoplasmic heterotrimeric G protein, leading to the modulation of downstream effector proteins [1,64]. Thus, GPCR-AAbs associated with chronic HF are of particular interest because their cardiac damaging effects are based on their impact on receptor-mediated autonomous heart regulation [65]. Evidence of GPCR-directed or GPCR-mediated humoral autoimmune cardiac injury pathogenesis was provided ex vivo and by experimental immunization, and it included aberrant activation of signaling pathways and receptor desensitization or hypersensitization, induction of cardiomyocyte apoptosis, and profibrotic stimulation of cardiac fibroblasts [1,65,66,67,68].

Among the about 800 receptors encoded in the human genome, the highly ligand-selective GPCRs, which constitute the largest receptor family, also play a key role in the regulation of the immune system [69,70]. It is assumed that every cell of the body expresses at least 15 to 20 GPCRs with relevant roles in the regulation of all body functions. GPCRs are widely expressed in immune cells, and notable examples in this regard are the adrenergic, cholinergic, and proteinase-activated receptors [69]. GPCRs are transmembrane proteins that are coupled to intracellular guanine nucleotide-binding proteins (G-proteins) that receive signals from outside the cell and transmit them internally for further biological processes [70]. The G-proteins, which are activated by their binding to guanosine triphosphate (GTP), trigger the production of various second messengers like cyclic adenosine monophosphate, which is crucial for cardiomyocyte function, or inositol triphosphate that mediates the release of intracellular calcium [70]. Thus, diseases that are characterized by the presence of GPCR-AAbs with evidence for disease-specific pathogenic activity could also be designated as “functional autoantibody disease” [63].

Since the 1970s, after the detection of AAbs in the serum of patients with DCM, several types of AAbs were found able to play a specific key role in the induction and progression of certain types of CM toward decompensated HF [3,71,72]. Among them, AAbs against cardiac myosin (cM) [72,73,74,75], β1-adrenergic receptor (β1AR) [71,72,76,77,78,79,80], M2 muscarinic acetylcholine receptor (M2R) [71,81,82,83,84,85], cardiac troponin I (cTnI) [85,86,87,88,89,90], and those against the angiotensin receptor 1 (AT1R), appeared of particular interest from clinical and pathological point of view [11,58,91]. Of these, certain AAbs like the β1AR-AAbs appeared mainly involved in the etiopathogenesis and progression of DCM [2,58,71], while others like the cTnI-AAbs appeared essentially involved in the clinical course and the prognosis of myocardial infarction-induced chronic HF [77,78]. In both idiopathic DCM and Chagas’ disease-related CM, the sera of the patients usually contain agonist-like AAbs directed against the β1AR and/or the M2R, which can affect their corresponding signaling cascades [71,85]. In a comparative study between a patient group with idiopathic DCM showing a β1AR-AAb prevalence ranging from 70% to 80% in their sera and another group with Chagas’ disease-related CM with a β1AR-AAb prevalence of only 29%, the β1AR-AAbs from the DCM group could be directed against amino-acid sequences of the first or the second extracellular loop, whereas those isolated from the Chagas’ CM patients recognized only one epitope on the second extracellular loop [71]. This indicates that even for the same type of AAb, there can be relevant differences in their epitope-recognizing abilities and also their pathogenicity, given that in the Chagas patient group, with the usually more severe CM, the prevalence was less than half that of the DCM. However, a causal link between non-ischemic HF and humoral autoimmunity against GPCR has not yet been completely established, except for Chagas’ CM [92]. One large study reported the presence of M2R-AAbs in 40% of patients with DCM, and those AAbs appeared highly predictive for atrial fibrillation [85].

A recent study revealed significant differences between the circulating AAb levels against the α1 adrenergic receptor (α1AR), β1AR, muscarinic acetylcholine receptor 5 (M5R), AT1R, and AT2R in HF patients and healthy controls, which remained significant upon correction for total IgG [92]. Among a broad panel of GPCR-AAbs analyzed simultaneously by comparable assays, only those five GPCR-AAb species levels were altered in the evaluated HF patients [92]. The abnormalities in the prevalence of those AAbs, which were equally distributed between histological sub-entities of non-ischemic HF classified as myocarditis, DCM, and other CMs (except Chagas’ CM), showed no correlation with the cardiac histological alterations [92]. Thus, although the alterations of the five GPCR-AAb types were associated with non-ischemic HF in a significant and possibly meaningful manner, the histological sub-entities allowed for no recognition of a distinct GPCR-AAb immunity profile [92]. It was also striking that not all the observed HF-associated alterations of GPCR-AAb levels pointed in the same direction. Thus, in HF patients, where the β1AR, M5R, and AT2R AAb levels were increased, the α1AR and AT1R AAbs were decreased [92]. However, given that current pathogenetic concepts propose a physiological regulatory role of GPCR-Abs that becomes imbalanced in autoimmune diseases, decreases in certain GPCR-AAbs could be likewise pathogenically relevant as the increases of others, given that current pathogenetic concepts propose a physiological regulatory role of GPCR-Abs that becomes imbalanced in autoimmune diseases [93,94]. The observed HF-associated alterations of AT1R-, α1AR-, and AT2R-AAbs, which reflect the effects of corresponding receptor activations in cardiovascular regulation, could also be relevant for HF due to their impact on arterial pressure regulation [92,95,96]. Additionally, the increased level of M5R-AAbs induced by systemic inflammation (triggered most often by periodontitis, especially in older persons), which is associated with receptor autoimmunization, can mediate myocardial dysfunction without inducing histological alterations [97].

### 3.2. Relevance of β1AR-AAbs in Dilative Cardiomyopathy

Functional AAbs with β-adrenergic agonistic-like effects on cardiomyocytes were isolated for the first time from sera of patients with idiopathic DCM already nearly 4 decades ago by Wallukat and Wollenberger [98]. Contrary to the physiologically regulated signal cascade, the binding of β1-AAbs to the β1AR leads initially to continuous overstimulation of the receptor without its down-regulation and desensitization of the ß-adrenergic reaction cascade [5,99]. Continuous hyperstimulation of β1ARs causes cardiomyocyte functional and structural damages associated with altered expression and sensitization of β1ARs (e.g., induction of mitochondrial dysfunction with intracellular Ca2+ overload, triggering of arrhythmias, provoking excessive myocardial fibrosis and cardiomyocyte apoptosis) [5,59,99,100].

There is strong evidence that AAbs stimulating the β1ARs can cause or lead to the progression of HF [101,102,103,104]. However, although there is no doubt about the involvement of β1AR-AAbs in the induction and progression of DCM toward chronic HF, there are several methodological aspects concerning the identification of functional β1AR-AAbs, which can create inconsistencies in the interpretation of certain study results and may also adversely affect the decision-making regarding the implementation of a β1AR-AAb removal therapy [101,102,103,104,105]. In this regard, major problems could arise from the questionable validity of peptide-based ELISA strategies in the diagnostics of cardio-pathogenic AAbs that activate GPCRs because detection of DCM-associated β1AR-AAbs requires functional readouts or native human β1AR as targets [63,103,104,105]. An important aspect, which can induce uncertainty or even mistakes in the evaluation of both the etiopathogenic importance of β1AR-AAbs and the therapeutic results provided by β1AR-AAb removal, is also the existence of 4 IgG subclasses (IgG1, IgG2, IgG3 and IgG4) of β1AR-AAbs according to the constitution of the fragment crystallizable region (Fc portion), which has different functionalities depending on their affinity to Fc receptors and their potency to activate effector cells [72,104]. A study that compared the IA therapy results obtained with the use of a protein-A column, which can remove IgG1, IgG2, and IgG4 but shows low affinity to IgG3, with those obtained with an anti-IgG column able to remove all IgG subclasses, revealed a more relevant improvement in LV ejection fraction (LVEF), cardiac index (CI), and stroke volume index [106]. Furthermore, in patients with DCM, IA on a protein-A column, when performed in conjunction with an improved treatment regimen for IgG3 elimination, was found superior to the sole use of that protein-A column-based conventional IA, in terms of both LVEF and CI improvement [107]. In studies using an optimized tryptophan column, which efficiently removes IgG3 subclass AAbs, the titer of IgG3 anti-myocardial AAbs (including β1AR-AAbs) appeared correlated with improvement in cardiac function after IA, whereas the titer of total IgG AAbs did not show such a correlation [108]. DCM patients with high gG3-β1AR-AAb levels responded more favorably to IA therapy [108]. All these results suggest that the IgG3 subclass could be more relevant to the pathology of DCM, and the pathogenetic roles of anti-myocardial AAbs, including β1AR-Aabs, could differ according to their IgG subclasses. Studies using β-blockers in HF patients suggest different therapy responsiveness and prognostic significance of β1AR-AAbs according to their IgG subclass. Thus, in patients with chronic systolic HF (LVEF < 40%), stable under predominant β-blocker therapy at inclusion into this study during 2.2 ± 1.2 years of follow-up, the presence at baseline of IgG3-β1AR-AAbs was associated with more favorable outcomes compared to those tested negative for IgG3-β1AR-AAbs, whereas the total amount of IgG-β1AR-AAbs appeared unable to differentiate patient outcomes [109]. That study also indicated that IgG3-β1AR-AAb-positive DCM patients can obtain important benefits from β-blocker therapy. Given that certain types of AAbs isolated from patients with DCM can induce a negative inotropy and reduction in calcium transients in vitro and ex vivo and that patients with cardio-depressant AAbs showed an acute improvement in cardiac function after IA therapy [110,111,112], the above mentioned more favorable outcomes of IgG3-β1AR-AAb-positive HF patients in comparison with those without evidence β1AR-AAbs suggest the existence of an important relationship between β-blockers and IgG3-β1AR-AAbs. Taken together, these findings suggest the possibility that IgG-β1AR-AAb subclasses could play different roles in the pathophysiology of CMs. Thus, as the authors assume, it is quite possible that the IgG3-β1AR-AAb subclass may exert a more direct pathological effect related to a primary autoimmune process like failure of self-tolerance, whereas non-IgG3-β1AR-AAbs could be more dependent on secondary autoimmune responses to self-antigens released as a result of cardiac damage [109].

Important details regarding the close connection between β1AR-AAbs and non-ischemic chronic CM (particularly DCM) were provided by the analyses of sera from patients with end-stage HF before and after ventricular assist device (VAD) implantation [4,6,113,114,115]. In an earlier study, 13 patients with end-stage idiopathic DCM tested positive for serum β1AR-AAbs revealed relevant LV improvement during mechanical support, which finally allowed for their weaning from that support [113]. Particularly remarkable was the disappearance (without any reappearance) of the β1AR-AAbs from the sera of all those patients during the VAD support [113]. The highly pathologic degree of fibrosis at the time of implantation also diminished during the VAD support and reached normal values about 1 year after explantation [113]. In another study, among 39 patients with chronic non-ischemic CM-induced HF supported by an LVAD as a bridge to HTx or as a permanent life-saving LV support and who could be weaned from that LVAD after weeks or months of support, revealed LV remodeling and functional improvement; 38 (97.4%) of them preoperatively tested positive for β1AR-AAbs [6]. In contrast to that, before LVAD removal, only one (2.6%) of the patients with evidence of LV reverse remodeling and functional improvement, which appeared sufficient for weaning the patient from the LVAD, remained further positive for β1AR-AAbs [6]. The disappearance of the β1AR-AAbs from the sera of the other 38 initially positive patients occurred during the first 2.6 ± 0.3 months of LVAD support and was nearly identical (*p* = 0.9) between the patients with and those without long-lasting (≥5 years) post-explant cardiac improvement [6]. Comparative histology revealed a regression of hypertrophy in 91% and a reduction in fibrosis in 64% of the weaned patients in whom that examination was possible [6]. Although it was evident that the presence of β1AR-AAbs before LVAD implantation was closely related to the LV injury, the stability of LVAD-promoted LV recovery also appeared significantly dependent on the preoperative severity of several structural and functional LV alterations, as well as on the stability of LV reverse remodeling and functional improvement [6]. Thus, incomplete LV reverse remodeling reflected by LV end-diastolic diameter > 55 mm, LV end-diastolic relative wall thickness (RWT) < 0.37, LV sphericity index > 0.67, LVEF < 45%, as well as the instability of LV size, geometry, and EF during short-time interruptions of the LVAD support and, particularly, a duration of HF beyond 5 years, were significant risk factors for post-weaning recurrence of HF [6,114,115,116,117]. Given that those studies included patients who received the best possible pharmacological treatment (including ß-blockers), the β1AR-AAbs deserve closer attention in the future in order to find additional treatments beyond the still important selective β1AR blockers, which are not sufficient.

Taking the aforementioned findings into consideration, there is hardly any doubt that inactivation and/or removal of functional β1AR-AAbs, already before the non-ischemic chronic CM-induced HF reaches its end-stage, can not only improve the patient quality of life but also provide important survival benefits for carefully selected patients. Elimination or neutralization of β1AR-AAbs can stop or even reduce the maladaptive ventricular remodeling and improve myocardial function and thereby also ameliorate the deleterious consequences of HF [4,59,61,72,107,109]. Given that a middle- or long-term LV mechanical support can reduce the β1AR-AAbs below pathologically relevant levels by reduction in the ventricular wall tension, thereby facilitating LV reverse remodeling and functional improvement, it appears to be very reasonable to remove these AAbs before the HF becomes in most cases irreversible. The latter would be incomparably less risky than a possibly necessary LVAD implantation, and the chances for improvement in cardiac function would also be higher.

Because of the limited and insufficiently convincing available data (mostly case reports) regarding the potential benefit of therapeutic plasma exchange for GPCR-AAb-positive patients, its use for β1AR-AAb-positive patients with DCM still cannot be recommended [1]. Quite different is the situation in the case of IA, which has emerged as a widely accepted therapy for β1AR-AAb elimination and has repeatedly proven its clinical value over nearly three decades [61,108,109,118,119,120,121]. More recently, it has also been demonstrated that intravenous application of small soluble molecules, such as peptides or aptamers, which specifically target and neutralize β1-AR-AAbs, could be, in certain cases, an alternative to extracorporeal IA [59,122,123,124,125]. Whereas peptides may induce immunogenicity, both animal and human studies with aptamers revealed no safety concerns and have demonstrated effectiveness in reducing AAb levels [61]. Certain aptamers with a large spectrum of action appeared to neutralize a variety of known circulating GPCR-AAbs [59,122,123,124,125].

Echocardiography is the major tool for the evaluation of LV function in patients with DCM-related HF, and LVEF is the most frequently used parameter for quantification of LV pump function. However, LVEF depends largely on the LV loading conditions (on preload and afterload), which indicates that the EF is not an index of myocardial contractility [126]. Given that in the presence of mitral regurgitation (MR), the difference between the LV end-diastolic and end-systolic volume is no longer the forward stroke volume (SVf) but becomes the sum of SVf and regurgitant volume, it is evident that MR can increase the volumetrically measured EF correspondingly with the increased blood volume leaving the LV during the systole [126]. Therefore, in advanced DCM, which is usually associated with a relevant secondary MR, the LVEF will become particularly unreliable as an individual parameter. Thus, an HF-induced aggravation of MR can maintain or even slightly increase the LVEF despite a reduction in the SVf, whereas a relevant reduction in MR can reduce the LVEF even in the presence of an improved SVf.

Although the speckle-tracking echocardiography-derived LV functional parameters are also not load-independent, due to their ability to differentiate myocardial wall motion from (displacement) myocardial contraction-induced circumferential and longitudinal shortening, as well as radial thickening of the myocardial walls, they provide much more reliable LV functional details especially in coronary artery diseases and DCM-related HF (particularly in the presence of relevant MR) [127].

Recently, by incorporating LV afterload estimations obtained by cuff blood pressure measurements into the global longitudinal strain (GLS) analysis, a new non-invasive method for evaluation of myocardial work has become an emerging tool in the evaluation of LV dysfunction by echocardiography, also for DCM-related HF patients [128]. This parameter has proven to provide less load-dependent measurements compared with GLS [128,129,130].

## 4. Immunadsorption for Patients with Non-Ischemic Cardiomyopathy

Even though β1AR-AAbs are not the only AAbs detectable in sera of patients with non-ischemic CM-induced HF, they often play the most relevant role in the triggering and progressive aggravation of HF, especially in idiopathic DCM-related HF [5,60,118,119,120,121]. One possible explanation could be the fact that the β1ARs, which are located on the cell surface, are easily accessible targets for AAbs in comparison with α-myosin or cTnI, which are immunologically sequestrated within the cells and, therefore, remain hidden from the immune system as long as they remain localized intracellularly [4,5,60,131]. The β1AR-AAbs also have a more pronounced cardiotoxic impact than the M2-muscarinic AAbs, which are often detectable in relevant quantities in the sera of patients with DCM [60,132].

Although, in in vitro and in vivo experiments, the β1AR-AAb-induced detrimental alterations of β1ARs associated with a reduction in β1AR-AAb-induced cardiotoxicity and cardiomyocyte apoptosis appeared reducible or partly even preventable by selective β1-adrenergic blockers like bisoprolol [3,76,98,133,134,135], and in clinical studies [106,136,137,138], selective β1-adrenergic blocker therapy can improve cardiac function and patient outcomes, the prognosis of β1AR-AAb-positive DCM is still poor, even under appropriate medical therapy. In many cases in which progression of HF cannot be stopped by pharmacotherapy, without the removal of the cardiotoxic AAbs, HTx remains the only therapeutic option. In view of the existing experimental and clinical data, treatment options that address the abnormalities of the humoral immune system appear very promising.

Removal of β1-AAbs can be achieved with extracorporeal IA by passing patients’ plasma over columns, which can provide either nonselective AAb binding (i.e., β1-AAbs together with AAbs belonging to other IgG subclasses, plus certain IgM- and IgA-AAbs) or more selective binding and removal of the whole IgG3-AAb subclass or only (truly selective) the β1AR-AAbs removal [61,117].

### 4.1. Beta1AR-AAb Immunoadsorption by Nonselective GPCR-AAb or Semi-Selective IgG3-AAb Removal

For “unspecific” (i.e., nonselective) or semi-selective IA with respect to the removal of β1AR-AAbs, most studies used either columns containing polyclonal anti-human Ig-Abs produced in sheep (TheraSorb^TM^ Ig adsorber) or columns containing synthetic peptide-based Ig adsorbers like tryptophan (i.e., Immunosorba TR-350) or the peptide-GAM (i.e., Globaffin) [1,61,118,119,120,121,139,140,141,142,143,144,145,146,147,148,149,150,151,152,153,154]. Compared with the TheraSorb columns, the latter two Ig adsorbers have a lower affinity for the AAbs belonging to the IgG3-subclass, which includes all the β1-AAbs [61]. After 2010, thanks to the introduction of a new IgG3-specific tryptophan column for IA therapy in patients with DCM-related advanced HF, this previously non-selective IA method became semi-selective regarding its ability to remove β1AR-AAbs [148]. In a pilot study, Wallukat et al. [118], nearly three decades ago, showed that for patients with DCM, unselective IA allowed for efficient removal of circulating β1AR-AAbs, associated with improvement in their functional NYHA class. Thereafter, several studies evaluating the short-term (up to 1 year) efficacy of unspecific IA in idiopathic DCM revealed significant improvement in LVEF and/or CI [120,139,140,141,142,143,144,145]. Short and long-term results of nonselective IA were also found to be better in β1-AAb-positive than in β1-AAb-negative patients [58]. In a large long-term follow-up study, which included 108 β1AR-AAb-positive DCM patients with chronic HF who underwent IA by using in 84 patients a TheraSorb column and 24 patients a Globaffin column, the probability for 5-year HTx/VAD-free survival reached 69.4% [61]. That survival rate was significantly higher than the HTx/VAD-free survival revealed in the same study by both β1-AAb-positive DCM patients without IA therapy (i.e., 25.5%) and β1-AAb-negative DCM patients who underwent the same IA (47.4%) [61]. Whereas in β1-AAb-positive DCM patients, the LVEF increased and the NT-ProBNP levels decreased significantly in both groups with different IA columns, β1-AAb-negative patients revealed no LVEF and/or NT-ProBNP improvement after IA [61]. During the first year after unspecific IA, the mean LVEF value increased in the whole β1-AAb-positive patient group, from 23.6% to 31.4% (i.e., 33% improvement *p* < 0.001) and in the subgroup of the 76 responders (i.e., those with evidence of LV functional improvement), from 23.5% up to 34.6% (i.e., over 47% improvement, *p* < 0.001) [61]. In the subgroup of responders, the 5-year HTx/VAD-free survival rate reached 89.3%, whereas in the non-responder subgroup, the latter reached only 24.2% (*p* < 0.001) [61]. After β1AR-AAb removal, the mean β1AR-AAb serum levels dropped from 5.3 ± 1.4 to 0.7 ± 0.8 LU (*p* < 0.001) and only in 8 (7.4%) of the 108 β1AR-AAb-positive patients, five IA sessions resulted in either none or insufficient β1AR-AAb reduction [61].

Another study compared the usefulness of unspecific IA using the broadband immuno-adsorber Globaffin (containing as ligand the synthetic peptide GAM, able to bind Igs independent from their Ag-specificity, but particularly IgG-class Abs) for treatment of non-diabetic and diabetic DCM patients with severe HF also confirmed the practical relevance of IA [119]. Thus, in both patient groups, there was a significant (*p* < 0.001) reduction in the prevalence of β1-AAb-positive DCM patients (from 77.4% to 9.7% in non-diabetic patients and from 80.6% to 6.5% in the diabetic DCM patient group) [119]. Thus, the massive post-IA reduction in the prevalence of β1-AAb-positive patients in the non-diabetic and diabetic patient groups reached -87.5% and -92%, respectively. Also important was the observation that the β1AR-AAb reappearance was relatively rare but in line with previous observations [61], but such reappearances appeared to be associated with worsening cardiac function. Post-IA, 3-year freedom from β1-AAb reappearance in patients with and without diabetes reached 72.1% and 71.1%, respectively, whereas the 3- and 5-year survival probabilities reached 81.3% and 52.9%, as well as 78.4% and 50%, respectively [119]. In all the evaluated DCM patients (i.e., 49 β1-AAb-positive and 13 β1-AAb-negative patients before IA), the prevalence of responders to IA (i.e., those with improved heart function and/or exercise tolerance) reached 67.7%. However, whereas the prevalence of responders to IA reached 79.6% in the initially β1-AAb-positive group, in the β1-AAb-negative patients before IA, the prevalence of responders to IA reached only 23.1% [119]. The post-IA LVEF improvement in the 31 diabetic DCM patients from 22.9% to 28.9% (+26%) and from 25.6% to 30.3% (+18%) in the 31 non-diabetic DCM patients were both statistically significant (*p* < 0.001) [119].

### 4.2. Selective Beta1-AAb Removal by Immunoadsorption

Specific (selective) IA can be performed with peptide columns (Coraffin, Affina) where the used peptides mimic the AAb-binding epitopes of the β1-adrenergic receptor, thereby allowing for selective elimination of β1-AAbs [149]. In a preliminary study including eight β1AR-AAb-positive patients with idiopathic DCM-related severe HF (LVEDD 69.3 ± 8.5 mm and LVEF 28.5 ± 6%) who underwent β1-AAb removal on five consecutive days, the β1-AAb levels decreased by 76% from 5.0 ± 0.5 laboratory units (LU) at baseline to 1.2 ± 0.6 LU after IA [149]. Simultaneously, there was also a significant reduction in LV size and improvement in LV function (LVEF increase up to 36.6 ± 10.7% and an increase in the tissue Doppler-derived LV systolic peak wall motion velocity with more than 74%). The autoantibody level decreased further and remained at 0.6 ± 0.6 LU (i.e., 88% lower than before IA) until the end of the first year after IA [149]. Ten years later, a large long-term follow-up study showed that selective IA using Coraffin columns could improve heart function in more than 78% of patients (referred to as “responders”) with end-stage idiopathic DCM, allowing for long-term cardiac stability with a 5-year survival probability of about 91 ± 6% without the necessity of a VAD implantation or HTx [61]. Patients who reached end-stage DCM, despite maximum drug therapy, could benefit from IA therapy to such an extent, whereas such patients showed neither benefits from intra-myocardial injection of autologous bone marrow mononuclear cells (BMNC) at LVAD implantation [150] nor conclusive evidence of benefits from intracoronary injection of BMNC [151,152,153]; this underlines the importance of β1-AAb removal, which is still the most efficient way to treat β1-AAb-positive patients, before the necessity of HTx or VAD support.

### 4.3. Comparison Between Unspecific and Specific Immunoadsorption for Beta1-AAb Removal

The efficiency of β1AR-AAb removal by non-selective or semi-selective IA appeared generally not inferior to that of selective (specific) IA. Thus, the reported prevalence of β1AR-AAb-positive patients with optimal β1AR-AAb removal by using TheraSorb (unselective), Globafin (semi-selective), or Coraffin (selective) columns was 84%, 81.6–87.5%, and 91.3%, respectively [61,119,147]. In a direct comparison, there were no significant differences between the prevalence of patients with optimal removal of β1AR-AAbs achievable by the above-mentioned IA methods [61,119]. The prevalence of “responders” to β1AR-AAb removal (i.e., improvement in both cardiac function and patient outcome) using either Therasorb, Globafin, or Coraffin columns reached 80%, 79–80%, and 78.3%, respectively [61,119]. The post-IA 5-year survival probability for patients who underwent IA using Therasorb, Globafin, or Corrafin reached 83.1%, 78.8%, and 91.3%, respectively. However, the differences did not appear statistically significant [61].

The important impact of β1AR-AAbs on the severity of HF in patients with DCM was particularly reflected by the significant difference observed between β1AR-AAb-positive and negative DCM patients, regarding the prevalence of responders to IA (i.e., improvement in cardiac function) on Globaffin columns that allowed for efficient removal of those AAbs in about 80% of the patients. Thus, the prevalence of responders appeared to be more than three times higher in β1AR-AAb-positive than in β1AR-AAb-negative DCM patients (i.e., 79.5% vs. 23.1%) [119].

## 5. Benefit-Risk Balance and Feasibility of IA Therapy for Patients with DCM-Related Heart Failure

Given that in β1AR-AAb with DCM associated with advanced HF, unselective, semi-selective, and selective IA appeared highly efficient for the relevant and stable reduction in β1AR-AAb serum levels associated with relevant improvement in cardiac function in up to 80% of those patients, as well as a relevant improvement in their long-term survival, the benefits of this therapy are undoubted. In severe DCM associated with diabetes mellitus (DM), where the prevalence of β1AR-AAbs can exceed 80%, the prevalence of responders to semi-selective IA on Globafin columns appeared to be even higher in comparison with non-diabetic DCM patients, and the 3-year post-IA HTx/VAD free survival was also found slightly higher in diabetic patients, comparable to that achievable after HTx [119]. Like in the DCM patients without DM, the 3-year and 5-year freedom from β1AR-AAb reappearance exceeded 74% and 63%, respectively [119]. Given that diabetic HF patients are generally at high risk for both pre-HTx and post-HTx mortality, the benefits from IA therapy are unequivocally evident. Thus, because IA appeared feasible in elderly patients and even in end-stage DCM associated with DM, whereas HTx or VAD implantation are incomparably more risky and also much more expensive, it would be quite reasonable to pay more attention to this effective therapeutic option [1,61,128]. Table 2 provides an overview of short- and long-term results of IA obtained in clinical studies, which included patients with DCM-related advanced HF.

In the guidelines of the American Society of Apheresis on the “Use of therapeutic apheresis in clinical praxis”, IA achieved a “grade B” (strong) recommendation with the remark that “IA can be applied without reservation in most patients in most circumstance” [154]. Also, in those guidelines, DCM is classified as a “class II” disorder for which IA is recommended as second-line therapy either as a stand-alone treatment or in combination with other treatment methods.

## 6. Conclusions

Despite many unanswered questions about the precise involvement of autoimmune mechanisms and the pathophysiologic relevance of different Abs against cardiac proteins in the induction and aggravation of cardiac morphological and functional alterations that characterize the idiopathic DCM, currently, there is no doubt about the important role played by the β1AR-AAbs in this context. The increasing prevalence of β1AR-AAb-positive patients with the aggravation of HF, reaching up to 97% in candidates for HTx or VAD implantation, as well as the high prevalence of HF patients with long-term improvement in both cardiac function and HTx/VAD-free survival after IA-induced normalization of β1AR-AAb levels, underline the key role of these AAbs. Moreover, the observation that β1AR-AAb-negative patients with DCM-related HF revealed, in most cases, no benefit from IA additionally emphasizes the implication of β1AR-AAbs in the majority of idiopathic DCM-related cases of advanced HF.

The unambiguous proof of the existence of a positive correlation between the prevalence of functional β1AR-AAb-positive DCM-associated HF patients and the severity grade of HF, as well as the high prevalence of cardiac responders (i.e., LV functional improvement associated with different degrees of reverse myocardial remodeling) to a successful AAb removal by IA therapy, strongly suggests the existence of a vicious circle between cardiac inflammation and β1AR-AAbs, which can be broken by IA therapy. Attesting to this are also the findings that the relatively rare reappearance of β1AR-AAbs was always associated with a renewed deterioration in LV function. Given that the probability of 3- and 5-year freedom from AAb re-increase can reach 75% and 64%, respectively, also speak in favor of a vicious circle sustained by β1AR-AAbs before their removal.

The good tolerability and the possibility to repeat the IA therapy not only in young persons but also in elderly HF patients, even in those with the coexistence of other chronic diseases (like DM), as well as the high responder rates to IA in β1AR-AAb-positive patients, are consistent with the guidelines of the American Society of Apheresis regarding the importance of this therapy for DCM-related potentially life-threatening HF.

## Figures and Tables

**Table 1:** Average prevalence of the different types of cardiac autoantibodies in healthy adults and patients with cardiomyopathy of different etiology.

Tested Adults	Average Prevalence (%) of the Different Types of Cardiac Autoantibodies			
	β1AR–AAbs	M2R–AABs	cMR–AABs	cTnI–AABs
Healthy persons	<10 (usually ≤1)	<17	<12.5	<13 (usually <10)
Ischemic CM	10–55 (usually 10–13)	50–62	4–30	10–21
DCM	26–80 (up to 97 in LVAD candidates)	15–50	20–66	7–22
Peripartum CM	60–100	46	24	26
Chagas’ CM	29–98	26–100	11–38	40–60

β1AR–AAbs: β1 adrenoreceptor autoantibodies; M2R: M2 muscarinic acetylcholine receptor; cTnI: cardiac troponin I; CM: cardiomyopathy; DCM: dilated cardiomyopathy. References nr. [58,59,60,61,62,63].

**Table 2:** Overview of short- and long-term results obtained in 11 clinical studies that included altogether 393 patients with DCM-related advanced heart failure associated with high β1AR-AAb levels who underwent immunoadsorption therapy for their removal.

Authors, Year, Ref.	Study Design, Type of IA	Baseline Characteristics	Efficiency of AAb Removal	Stability of Post-IA AAb Levels	Prevalence of Responders to IA	Improvement in Cardiac Function	Short- and Long-Term Post-IA Outcome
Dörffel et al., 1997 [139]	- NS-IA (Ig-Therasorb^®^) in 9 patients with DCM - After the last IA session, patients received an infusion of polyclonal IgG to restore the IgG serum levels.	- NYHA III or IV - LVEF < 25% - β1-AAb (+)	- IA highly effectively reduced the β1-AR-AAbs in all patients, with an average decrease from 6.4 to 1.0 relative units (rU).	- Stability of post-IA AAb levels was not investigated.	- Prevalence was not analyzed. The proven hemodynamic benefits of IA might suggest a high prevalence.	- Significant CI and SVI increase from 2.0 to 2.9 L/min/m^2^ and from 24 to 36 mL/m^2^, respectively. - LVEDP, PCWP, and PAP also decreased (*p* < 0.01).	- Short-term post-IA outcome was good in all patients. - In all patients, IA was well tolerated, with no major complications.
Müller et al., 2000 [120]	- CC prospective study - NS-IA (Ig-Therasorb^®^) in 17 patients (one course with 5 sessions/17 conventionally treated (controls).	- NYHA II-IV - LVEF < 30% - β1-AAb (+)	- IgG drop below 120 mg/dL was demonstrable in all patients. - β1-AAb level normaliszation (reduction from 5.0 to <1.0 LU, *p* < 0.001) in all patients.	- After further reduction during the first 3 months, the β1-AAb levels remained stable in the normal range until the end of the 1st year.	- All 17 patients who underwent IA responded to the therapy.	- LVEF increased on average from 25% to 38%. - LVEF and NYHA class improvements reached a *p* < 0.001.	- At the end of the 1st year, all patients were stable with an improved heart function, whereas in the control group, 2 patients underwent HTx.
Felix et al., 2000 [140]	- RCT, 18 patients with DCM - NS-IA (Ig-Therasorb^®^) in - 9 patients/9 controls - Initially, one IA session on 3 consecutive days with IgG substitution thereafter - IA repetition at one month.	- NYHA III or IV—clinically stable - LVEF < 30%	- During the first IA course, IgG and β1-AAb serum levels decreased significantly from 10.9 to 2.2 g/L and from 4.8 to 0.38 relative U (rU), respectively.	- After the 1st IA course, the β1-AAb levels increased on average to 3.3 rU before the 4th IA course. - After the 4th IA course, the β1-AAb levels decreased below 0.5 rU.	- The prevalence of responders was not reported, but the available data suggest a high prevalence.	- Significant CI, SVI, and LVEF increase in the IA-group from 2.1 to 2.8 L/min/m^2^, from 27.8 to 36.2 mL/m^2^, and from about 23% to 34%, respectively.	- IA was well tolerated by all patients, and there were no major complications.
Felix et al., 2002 [141]	- CC prospective study - NS-IA (Ig-Therasorb^®^) in - 11 DCM patients/9 controls - One session/day on 3 consecutive days with subsequent IgG substitution.	- NYHA III/IV - LVEF < 30% - CI < 2.5 L/min/m^2^	- IgG and β1-AAb serum levels decreased significantly from 10.7 to 2.4 g/L and from 4.4 to 0.3 rU, respectively. - The CE revealed depressant effects on isolated cardiomyocytes.	- The further course of IgG and β1-AAb serum levels was not investigated.	- The prevalence of responders was not reported.	- Significant CI and SVI increase after IA from 2.2 to 2.7 L/min/m^2^ and from 31 to 37 mL/m^2^, respectively, which were similar to those observed by the authors in a previous study.	- Like in a previous study, IA was well tolerated by all patients, and there were no major complications.
Staudt et al., 2002 [106]	- CC prospective study - NS-IA in 18 DCM patients, 9 on protein A columns and 9 on anti-IgG columns (Plasma-Select) with subsequent IgG substitution.	- NYHA III or IV—Clinically stable - LVEF < 30%, - CI ≤ 2.5 L/min/m^2^ - Duration of the disease > 2 years	- IgG level decreased after IA in both groups, by −83% and −85%, respectively. - The β1-AAb levels decreased in the anti-IgG group from 4.8 to 1.2 uU (*p* < 0.01 versus baseline and versus protein A group).	- In the anti-IgG group, 3 months later, the β1-AAb levels reached only 1.1 rU (normal range), whereas in the protein A group, their level was 3 times higher.	- The prevalence of responders was not reported, but the available data indicate a high prevalence.	- After 3 months, whereas LVEF did not significantly increase, in the protein A group, there was a significant LVEF increase from 21% to 31% in the anti-IgG group (*p* < 0.01).	- All patients tolerated IA and subsequent IgG substitution well, without complications (including any signs of infections).
Baba et al., 2010 [109]	- 18 patients with DCM - IA on a selective IgG3 ad-sorption column, 3–5 times/course for β1-AAb and/or M2R-AAb removal.	- NYHA III or IV - LVEF < 30% - 16 patients were tested positive for β1-AABs.	- β1AR- and M2R-AAbs were completely removed by IA; - 15 (83.3%) tested negative for all cardiodepressant AAbs after the 1st IA course.	- In one patient, the β1AR-AAbs reappeared early after IA and reached after 1 year 40% higher levels than initially. After the second year, the patient died of HF.	- The prevalence was only 44.4% due to the large group of patients with incomplete treatment.	- After 3 months, in the complete treatment group, LVEF improvement reached, on average, 53% (*p* < 0.01).	- IA was well tolerated and without relevant side-effects. - Cardiac function and quality of life improved during the first 3 months after IA.
Dandel et al., 2012 [61]	- RA included 108 patients with DCM (NS-IA on Thera-Sorb or Globaffin columns between 1995 and 2005). - S-IA for other 24 DCM patients on Coraffin columns.	- HTx candidates with LVEF < 30%. - Prevalence of β1-AAB-positive patients reached 82%.	- The efficiency of NS-IA reached 93.5%. In 2 patients, there was no reduction, and in other 5, there was no relevant reduction in β1-AAb levels. - The S-IA efficiency reached 91%.	- During the first 3 post-IA years after NS-IA, β1AR-AAbs were redetected in 14.8% of the patients. - β1-AAb reappearance had negative consequences in 76% of those patients.	- A total of 79.6% responded to NS-IA. No differences regarding the column used for IA. - In the S-IA group, that prevalence was 78.3%.	- LVEF increased on average from 23.5% to 34.6% (i.e., about 47% increase). - Prevalence of NYHA class ≥ III among the responders dropped from 89.5% to 51.3% (*p* < 0.001).	- The 5-year HTx/VAD-free survival probability reached 69.4%, whereas, in β1-AAb-positive patients without IA, that probability reached only 25.5%.
Yoshikawa et al., 2016 [108]	- Prospective study, 43 patients with DCM-related refractory HF underwent SS-IA on optimized tryptophan columns (Immunsorba TR) for removal of IgG3 subclass AAbs.	- 95% NYHA III, - 5% NYHA IV - LVEF < 30%	- IgG3 and β1-AAb elimination rates reached 98% and 95%, respectively.	- There were no published data about the potential reappearance of AAbs.	- During the first 3 months post-IA, the LVEF increased in 22%, and NYHA improved in 52% of the treated patients.	- Echo-derived LVEF, but not radionuclide LVEF, has improved after IA. - VO_2_ max and 6 min WD improved significantly (*p* = 0.006 and *p* = 0.005, respectively).	- Most of the adverse events during the IA were mild and easily controllable. - Cardiac events occurred more frequently in the first 3 months of this study than later, between the 6th and 12th month.
Dandel et al., 2015 [119]	- RA included patients with DCM-related HF, 31 with and 31 without additional type 2 DM who underwent the same NS-IA on Globaffin columns.	- LVEF ≤ 30% - β1-AAB-positive in the group with and without DM: 77.4% and 80.6%. - DM patients were older and with longer history of HF.	- A total of 90% of all β1-AAb-positive DCM patients (with and without DM) became negative after IA. - A total of 92% of the β1-AAb-positive DCM patients with additional DM became negative after IA.	- The probability of 3- and 5-year freedom from AAb re-increase was estimated at 75% and 64%, respectively. - Post-IA freedom from β1-AAb reappearance in those with and without additional DM was 72.1% and 71.1%.	- Prevalence of responders was 68% - In β1-AAb-positive patients before IA, the prevalence of responders was 79.6%.	- In responders with and without coexistent DM, the LVEF increased on average from 23% to 33% (43.5% improvement)	- The 3- and 5-year HTx/VAD-free survival probabilities for all 64 evaluated patients reached 79.6% and 63.5%, respectively. - The 3-year survival probability for DCM patients with and without DM reached 81.3% and 78.4%, respectively (*p* > 0.05).
Ohlow et al., 2016 [146]	- Prospective case study; 89 DCM patients - NS-IA on protein columns (Immunsorba) for IgG3 reduction, followed by polyclonal IgG substitution.	- NYHA III or IV - LVEF ≤ 40%	- No Ig quantification neither before nor after IA therapy.	- Not investigated	- Only 48% of the patients were classified as responders.	- The LVEF increased on average from 30% to 38% (+27% improvement) - In the non-responder and responder group, the LV EF increased from 30% to 35% (+17%) and from 27% to 40% (significant, + 48% improvement).	- There was a relatively high (17.2%) complication rate (e.g., pneumothorax in 2 patients, 5 allergic reactions, and 9 minor local infections at the catheter entry site). - Zero mortality during the 12-month follow-up.
Cvusoglu et al., 2023 [147]	- Prospective case study; - 9 DCM patients tested positive for β1AR-AAbs underwent NS-IA therapy on tryptophan columns (1 course in 5 successive days with 1 session/day) with subsequent polyclonal IgG substitution.	- NYHA III–IV - LVEF < 30%. - All received β-blocker therapy.	- No AAb quantification after IA therapy.	- Not investigated.	- The prevalence of responders was not reported, but the extent of cardiac improvement indicates a high prevalence.	- The LVEF increased on average during the first 6 months from 23% to 35% (52% increase; *p* = 0.01), and the NTproBNP decreased, on average, from 1161 to 385 pg/mL (33% reduction, *p* = 0.04).	- All patients remained free from any major complications. No adverse event was observed during and after IA therapy.

DCM: dilated cardiomyopathy; β1AR-AAbs (also abbreviated as β1-AAbs): β1 adrenoreceptor autoantibodies; IA: immunoadsorption; NS-IA: non-specific IA; Ig: immunoglobulin; LVEF: left ventricular ejection fraction;CI: cardiac index; SVI: stroke volume index; LVEDP: LV end-diastolic pressure; PCWP: pulmonary capillary wedge pressure; PAP: pulmonary arterial pressure; CC: case-control; HTx: heart transplantation; RCT: randomized controlled trial; S-IA: specific immunoadsorption; CE: Column eluent; M2R: M2 muscarinic acetylcholine receptor; VAD: ventricular assist device; HF: heart failure; RA: retrospective analysis; VO_2_ max: maximal oxygen consumption; DM: diabetes mellitus; NTproBNP: N-terminal pro-b-type natriuretic peptid; WD: walking distance.

## Abbreviations

HF = heart failure; IA = immunoadsorption; LV = left ventricle; AAbs = autoantibodies; IgG3 = subclass of immunoglobulins, which includes the β1AR-AAbs; STE = speckle tracking echocardiography.
